# Development and validation of an accurate smartphone application for measuring waist-to-hip circumference ratio

**DOI:** 10.1038/s41746-023-00909-5

**Published:** 2023-09-11

**Authors:** Siddharth Choudhary, Ganesh Iyer, Brandon M. Smith, Jinjin Li, Mark Sippel, Antonio Criminisi, Steven B. Heymsfield

**Affiliations:** 1 Amazon Inc., Seattle, WA USA; 2 Pennington Biomedical Research Center, Louisiana State University System, Baton Rouge, LA USA

**Keywords:** Computer science, Risk factors, Body mass index, Obesity, Diagnostic markers

## Abstract

Waist-to-hip circumference ratio (WHR) is now recognized as among the strongest shape biometrics linked with health outcomes, although use of this phenotypic marker remains limited due to the inaccuracies in and inconvenient nature of flexible tape measurements when made in clinical and home settings. Here we report that accurate and reliable WHR estimation in adults is possible with a smartphone application based on novel computer vision algorithms. The developed application runs a convolutional neural network model referred to as MeasureNet that predicts a person’s body circumferences and WHR using front, side, and back color images. MeasureNet bridges the gap between measurements conducted by trained professionals in clinical environments, which can be inconvenient, and self-measurements performed by users at home, which can be unreliable. MeasureNet’s accuracy and reliability is evaluated using 1200 participants, measured by a trained staff member. The developed smartphone application, which is a part of Amazon Halo, is a major advance in digital anthropometry, filling a long-existing gap in convenient, accurate WHR measurement capabilities.

## Introduction

More than seven decades ago, in 1947, the French professor of medicine Jean Vague first reported body shape phenotypes associated with the metabolic derangements of obesity^1^. Vague’s seminal observations were carried forward in the early 1980s by Krotkiewski and his colleagues who associated metabolic disturbances with regional adipose tissue deposits and fat cell size and number^2^. Men with obesity, according to the investigators, had a high-risk adipose tissue distribution characterized by abdominal obesity compared to women whose adipose tissue was located primarily in the gluteofemoral region. The high-risk abdominal obese phenotype was characterized, independent of sex, by a high waist-to-hip circumference ratio (WHR). The following year Larsson et al. found in a 12-year follow-up study of men that abdominal obesity, characterized by a large WHR, was associated with an increased risk of myocardial infarction, stroke, and premature death independent of generalized obesity as defined by body mass index (BMI)^3^. WHR soon became recognized as an index of intra-abdominal and subcutaneous adipose tissue distribution^4^. These early observations prompted a World Health Organization (WHO) Expert Consultation in 2008 that critically reviewed technical measurement and clinical aspects of both waist circumference and WHR^5^. Of the many biometrics for characterizing the health risks of excess adiposity, the WHR consistently ranks as the best or one of the best predictors of disease outcomes^6–10^. Our group recently introduced a calculus-derived, normalized sensitivity score to compare the predictive power of diverse adiposity biomarkers^11^. Our findings, using the National Health and Nutrition Examination Survey (NHANES) database, again confirmed, among the multiple available adiposity biomarkers, that WHR has the strongest associations with the risks of common health conditions. Despite these findings, extending now over several decades, WHR is rarely measured in clinical or home settings. One reason is that healthcare workers and people with obesity are not well trained on the nuances of anthropometric measurements as recommended by the WHO and other health organizations. Sebo and colleagues conducted extensive studies of the anthropometric measurement skills of primary care physicians^12^. Even with training, measurement error was consistently highest for WHR and lowest for weight and height^13,14^. The potential value of WHR as a health risk biomarker is thus not being realized outside of specialized research laboratories and clinical facilities.

Recent developments in computer vision now have the potential to transform the measurement of biometrics, including WHR. Our group has introduced a smartphone application that is highly accurate and reproducible in quantifying a person’s anthropometric dimensions, including circumferences, lengths, surface areas, and volumes^15,16^. The possibility thus exists to accurately estimate WHR using a smartphone application based on computer vision algorithms.

Here we report that accurate and reliable WHR estimation in adults is possible with a smartphone application based on novel computer vision algorithms. The application analyzes color images taken from various angles and employs a Convolutional Neural Network to predict body circumferences and WHR. This bridges the gap between clinical measurements by professionals and often inconsistent self-measurements at home. We validate MeasureNet’s accuracy and reliability with 1200 participants, all measured by a trained staff.

## Results

Over 1200 participants were evaluated in the current study (Supplementary Note 3). The CSD dataset included 270 men and 280 women, the Human Solutions dataset included 215 men and 326 women, and the noise evaluation sample included 71 men and 83 women. The demographic characteristics of these samples are summarized in Supplementary Note 9. Overall, the datasets included a range of race/ethnicities and average BMIs were in the overweight range. Men had average WHRs that were larger than those in women (~0.90 vs. 0.85). WHR measurements range for CSD is 0.877 ± 0.201 and for Human Solutions dataset, it is 0.857 ± 0.240.

### Accuracy

**Fig. 1:**
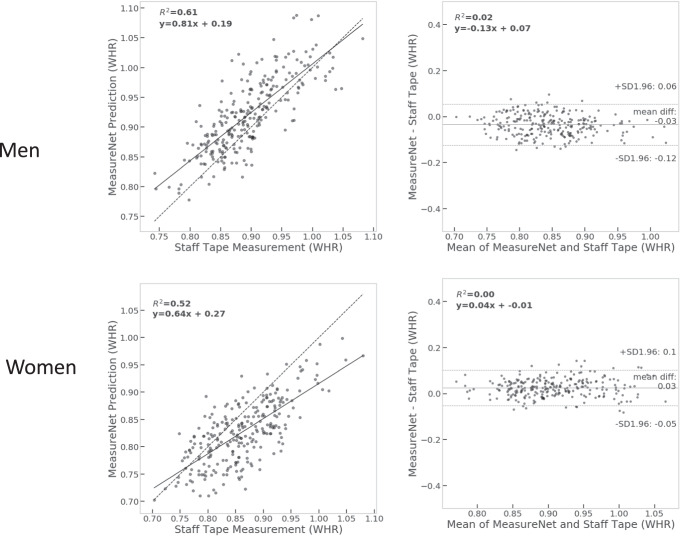
Quantitative comparisons. (left) Correlation between WHR measured by a trained staff member and WHR predicted by MeasureNet. The dashed line is identity and the solid line is the fitted regression line. (right) Bland-Altman analyses of the differences between WHR measured by a trained staff member and WHR predicted by MeasureNet. The horizontal dashed black lines are at mean ± 1.96 standard deviations.

The accuracy estimates for MeasureNet and self-measured WHR are presented in Table 1. MeasureNet’s MAE and MAPE estimates were about one half those of self-measured WHR (~0.015 and 1.4% vs 0.025 and 2.8%). Correlation between WHR measured by a trained staff member and WHR predicted by MeasureNet is shown in Fig. 1.

**Table 1:** Accuracy of MeasureNet and self-measured WHR estimates.

Sex	*N*	MeasureNet			Self-measured		
		MAE	MAPE (%)	P90	MAE	MAPE (%)	P90
Men	270	0.0122	1.34	0.0406	0.0259	2.79	0.0728
Women	280	0.0169	1.35	0.0363	0.0239	2.87	0.0624

Accuracy is measured relative to ground truth staff measurements using mean absolute error (MAE), 90th percentile error (P90) and mean absolute percentage error (MAPE). Lower is better.

### Comparison with state-of-the-art approaches

**Fig. 2:**
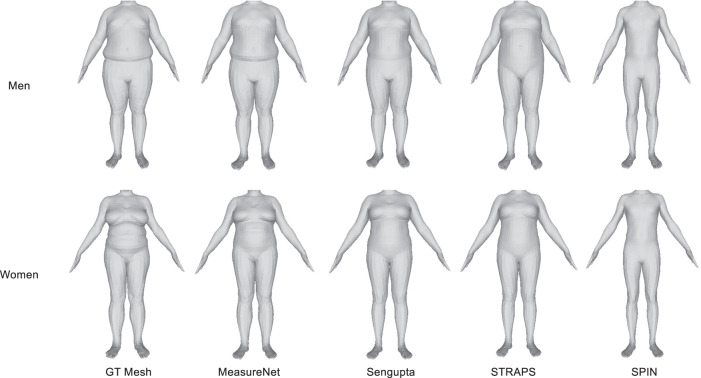
Qualitative results. Qualitative comparisons of the SMPL mesh predicted by MeasureNet and state-of-the-art approaches for 3D human shape and pose estimation^17,20,21^. Images correspond to results presented in Tables 2 and 3. The ground truth (GT) mesh is shown in the left.

The direct SMPL mesh-based predictions are compared in Tables 2 and 3. MeasureNet, with semantically segmented three views (front, side and back) as input and direct prediction, had the lowest MAE. Using three views as input to MeasureNet had lower error than using only the front view or front and side view. Using direct prediction had lower error than first reconstructing the body model and then extracting measurements from it. Direct prediction allows the measurement of each body part to be independent of the space of global SMPL parameters and results in better prediction of subtle body shape details. Additionally, using a silhouette as input to MeasureNet increased prediction error as compared to using a segmentation image as input. For Sengupta et al.^17^, we compared sex-specific and sex-neutral models and we found that the sex-specific model had lower error than sex-neutral model. Sex-specific model uses different model for different sex allowing the model to learn unique features for each sex. Sex-neutral model uses the same model for both the sexes. Qualitative comparisons between predicted and ground truth meshes are shown in Fig. 2. As seen from the figure, MeasureNet’s predictions are more accurate when compared to other approaches and have more accurate prediction of fat folds near the torso. Head images were cropped for privacy reasons. Additional comparisons for men and women are shown in Supplementary Figs. 5 and 6, respectively.

**Table 2:** Comparisons of MeasureNet with state-of-the-art approaches for estimating body circumferences for men (MAE, mm).

Method	Hip	Waist	Chest	Thigh	Calf	Bicep
SPIN^20^	104.71	128.34	162.48	71.84	32.22	69.73
STRAPS^21^	73.59	66.36	50.25	31.34	24.84	21.74
Sengupta et al. (Sex-neutral model)^17^	45.88	47.13	60.83	30.57	19.65	25.56
Sengupta et al. (Sex-specific model)^17^	39.13	42.78	50.76	25.92	18.18	22.72
Smith et al.^30^	24.04	25.56	25.85	17.33	15.96	11.32
MeasureNet (1 view + Sil + SMPL)	31.80	35.90	33.19	21.61	16.63	11.48
MeasureNet (1 view + SMPL)	25.36	28.43	29.49	17.86	15.38	10.29
MeasureNet (2 views + SMPL)	24.70	25.75	28.86	15.79	13.83	11.95
MeasureNet (3 views + SMPL)	23.83	24.83	24.38	14.40	11.88	9.99
MeasureNet (1 view + Direct)	17.72	23.25	22.86	14.02	13.18	8.96
MeasureNet (2 views + Direct)	15.97	17.47	17.83	12.04	12.02	9.44
MeasureNet (3 views + Direct)	14.38	16.38	15.90	11.69	9.67	7.95

One view (front view), two views (front and side) and three views (front, side and back) are the number of input views to MeasureNet. SMPL or Direct is the circumference prediction method. “Sil” uses silhouette as input. Sample size is 215 men. Lower is better.

**Table 3:** Comparisons of MeasureNet with state-of-the-art approaches for estimating body circumferences for women (MAE, mm).

Method	Hip	Waist	Chest	Thigh	Calf	Bicep
SPIN^20^	106.59	119.13	116.07	66.74	26.01	40.57
STRAPS^21^	90.78	82.31	73.34	43.10	28.97	28.75
Sengupta et al. (Sex-neutral model)^17^	51.64	44.53	42.56	29.74	18.33	18.34
Sengupta et al. (Sex-specific model)^17^	40.05	40.01	41.18	31.13	18.16	21.21
Smith et al.^30^	36.74	42.10	38.00	21.57	15.89	15.23
MeasureNet (1 view + Sil + SMPL)	40.50	36.14	38.95	17.57	12.54	11.12
MeasureNet (1 view + SMPL)	32.62	35.20	36.85	18.66	14.66	11.11
MeasureNet (2 views + SMPL)	31.10	26.95	35.80	20.02	12.95	10.74
MeasureNet (3 views + SMPL)	26.48	28.70	28.64	17.19	11.08	10.60
MeasureNet (1 view + Direct)	23.07	28.91	25.44	15.12	11.19	9.73
MeasureNet (2 views + Direct)	21.91	24.03	22.30	11.66	8.85	9.14
MeasureNet (3 views + Direct)	14.43	21.67	20.07	11.07	7.14	8.16

One view (front view), two views (front and side) and three views (front, side and back) are the number of input views to MeasureNet. SMPL or Direct is the circumference prediction method. “Sil” uses silhouette as input. Sample size is 326 women. Lower is better.

### Measurement noise

The noise distributions of MeasureNet predictions, self-measurements, and trained staff-measurements are plotted as histograms in Supplementary Figs. 7–9. The noise standard deviations are shown in Table 4. The standard deviation of noise in self-measurements was larger compared to MeasureNet and trained staff measurements. The smallest standard deviations, and thus noise, were for MeasureNet for both the men and women.

**Table 4:** Noise distributions (standard deviations, mm) of staff-measurements, MeasureNet predictions, and self-measurements.

	Hip	Waist	Chest	Thigh	Bicep	WHR
Men						
Self-measured	24.72	23.69	24.05	27.07	19.40	0.033
Staff-measured	14.48	12.94	14.90	15.07	9.99	0.015
MeasureNet	0.01	0.01	0.01	0.005	0.003	0.011
Women						
Self-measured	19.46	34.77	26.75	27.10	18.05	0.036
Staff-measured	11.84	15.02	13.93	16.35	8.82	0.017
MeasureNet	0.008	0.010	0.009	0.006	0.005	0.010

Sample sizes are 73 men, 83 women. WHR is waist to hip ratio.

### Synthetic dataset evaluations

The accuracy and repeatability for MeasureNet’s predictions compared to synthetic data ground truth are presented in Table 5. MeasureNet’s accuracy MAE for body circumferences (hip, waist, chest, thigh, bicep) on the synthetic dataset is lower than the accuracy MAE on the Human Solutions dataset (Tables 2 and 3). This is due to the remaining synthetic-to-real domain gap between training (synthetic meshes) and test distributions (laser scanned meshes). As we improve the realism of synthetic training data, we expect this gap to reduce further. MeasureNet’s WHR accuracy MAE is lower on the synthetic dataset compared to the accuracy MAE on the CSD dataset (0.0079 on synthetic vs 0.0122 on CSD for men, 0.0078 on synthetic vs 0.0169 on CSD for women). This is due to the combination of synthetic-to-real domain gap and the measurement noise in tape measured ground truth.

**Table 5:** Accuracy and repeatability of MeasureNet predictions on synthetic data.

	Accuracy		Repeatability	
Men	MAE	P90	MAE	P90
Hip	6.96	15.34	5.39	11.53
Waist	7.22	16.10	5.29	11.14
Chest	6.24	13.41	5.03	10.58
Thigh	4.45	9.76	3.50	7.41
Bicep	4.04	9.15	2.81	5.98
WHR	0.0079	0.0175	0.0057	0.0127
Women				
Hip	7.11	16.00	5.09	10.89
Waist	7.69	16.12	5.82	12.02
Chest	7.13	15.40	5.66	12.20
Thigh	5.20	11.62	3.82	8.25
Bicep	3.92	8.46	3.01	6.44
WHR	0.0078	0.0166	0.0058	0.0120

Body part circumference (hip, waist, chest, thigh, bicep) accuracy and repeatability are measured in mm. Sample sizes are 100 men, 100 women. WHR is waist to hip ratio.

## Discussion

The current study confirms that accurate and reproducible estimates of the WHR can be acquired with a smartphone application. Specifically, our developed MeasureNet application provided WHR estimates with respective MAEs and MAPEs of ~0.012–0.017 and 1.3–1.4% relative to those of flexible tape measurements made by skilled technicians, used as ground truth. These MAEs and MAPEs were less than half those of self-measurements. These proof-of-concept observations, the first of their kind, indicate that smartphone applications such as MeasureNet can now fill the void in WHR measurements made in clinical and home settings. The smartphone approach can potentially displace 3D scanning methods^18^ that are more costly and impractical to implement outside of specialized research and clinical facilities.

Human shape and pose estimation are active areas of research in the computer vision and machine learning (CVML) communities. Most of the current approaches predict body shape using a learned model or fit body shape using an optimization-based approach with SMPL^19^, a parametric 3D body model given observations such as 2D key points, silhouettes, or images^20–24^. Recent developments as reported by Sengupta et al.^17^ are the closest to our current approach as the investigators used synthetic data to learn human pose and shape estimation networks. In contrast to their approach, we focused directly on estimating body circumferences and derived measures such as the WHR, a strategy we found more accurate than estimating body circumferences from the reconstructed body model. Our MeasureNet model estimates circumferences and the WHR directly, and uses SMPL, the parametric 3D body model only as a regularizer during training. This allowed the circumference predictions to be independent of the space of SMPL parameters. Several challenges needed to be overcome on the path to developing MeasureNet. First, MeasureNet needed to generalize to different body shapes and be invariant to lighting and background conditions, clothes worn, user distance from the smartphone, and smartphone type. Our MeasureNet algorithms account for all of these factors and conditions that became apparent during the software development phase. Another factor posing a development challenge was that training accurate CVML models required access to accurate ground truth measurements. Manual measurements of waist and hip circumferences, however, tend to be error prone as reported by Sebo et al.^12,14^ and in the current study (Table 4). On the other hand, using highly accurate 3D laser scanners to extract ground truth measurements is expensive and time consuming. We addressed both problems by training a CNN on realistic-looking synthetic data sampled according to an empirical distribution, and we demonstrated strong generalization (high accuracy and repeatability) to real, previously unseen test images. Adding WHR estimates to clinical and self-evaluations improves health risk predictions beyond those of BMI and other currently available biometrics^11^. The underlying mechanism appears to be captured by the WHR of an individual’s body shape as defined by the sizes of their visceral and gluteofemoral adipose tissue depots. Larger visceral adipose tissue volumes and waist circumferences are associated with greater risks of adverse health outcomes^7–10^. By contrast, larger subcutaneous gluteofemoral adipose tissue volumes and hip circumferences are associated with a reduced risk of developing multiple cardiovascular and metabolic outcomes^6^. Their combination in the WHR thus is a sensitive body shape phenotype that establishes a person’s health risks. Smartphones or similar devices capable of generating two-dimensional images can thus be used to classify a person’s shape risk phenotype in clinical and even home settings; changes with aging or interventions can be tracked over time. WHR or the individual waist and hip circumferences can also be added to health outcome prediction models now in development by our group and others. Large-scale studies designed to identify health-risk genetic markers can use programs like MeasureNet to accurately capture participant shape using their own smartphones. Anticipated camera advancements and future machine learning algorithm refinements over time will further expand the applicability of smartphone phenotyping methods.

There are several limitations with our developed model that form the potential basis of future research. As part of the realistic sampling process the current SMPL 3D mesh model was estimated using 3500 3D scans covering the US general population and therefore is biased towards the average North American population. This kind of potential bias can be removed by including 3D scans of participants outside of the US when estimating the SMPL 3D mesh model. Future studies with ground truth estimates are needed to further define MeasureNet accuracy and reproducibility in “real world” settings.

A subset of participants In the CSD dataset had only one measurement taken by trained clinical staff. Therefore, the resulting ground truth measurement can be noisy and it can affect the accuracy metrics. A larger scale study where each participant is measured by multiple trained clinical staff members and includes 3D scanner ground truth can be useful to further validate MeasureNet’s accuracy and robustness as compared to tape measured ground truth.

The MeasureNet model is trained using synthetic training data. However, the current synthetic data generator can only represent the shape and pose of a minimally clothed body and fails to model complex topology of loose clothing. This results in a synthetic-to-real domain gap that reduces the accuracy of MeasureNet. A more realistic synthetic data generator that can model loose clothing can help alleviate this issue.

Another limitation of this study is that no research has yet been conducted to investigate the relationship between MeasureNet’s predictions and health risks. Further studies to understand the relationship between MeasureNet and health risks can help determine the desired accuracy level needed for an accurate health risk prediction.

Human Solutions dataset has a racial bias since it predominantly consists of 90% of individuals from Black and White racial groups, with limited representation from other races. Addressing this bias requires the inclusion of participants from underrepresented groups to foster a more balanced and equitable dataset.

In conclusion, the current study fills a long-held gap in accurately and reproducibly quantifying the WHR, an extensively researched health-risk biometric, outside of specialized facilities. The developed novel software, MeasureNet, can operate on conventional smartphones and thus vastly extend shape phenotyping capabilities to a large percentage of the global population, even to remote settings. Future studies are needed to extend software capabilities to populations beyond those in North America and to non-adult age groups.

## Methods

### Experimental design

The study hypothesis was tested in two phases. A smartphone application based on computer vision algorithms was developed in the first study phase. The development of this algorithm, MeasureNet, is described in the methods section that follows.

The second phase involved testing MeasureNet performance in a series of experimental studies (Supplementary Fig. 3). First, the accuracy of MeasureNet and self-measurements were compared to flexible tape measurements taken by trained staff in a sample of healthy adults referred to as the *Circumference Study Dataset* (CSD). Accuracy metrics are defined in the Statistical Methods section. Front-, side-, and back-view images of users were collected with a smartphone along with “ground truth” flexible tape circumference measurements taken by trained staff and by the user themselves. Circumferences were measured according to NHANES guidelines (Supplementary Note 1). MeasureNet and self-measurements were compared to the ground truth tape measurements.

A second experimental study involved comparison of MeasureNet to state-of-the-art approaches for three-dimensional (3D) shape estimation. Specifically, we compared MeasureNet, SPIN^20^, STRAPS^21^, and recent work by Sengupta et al.^17^ to ground truth estimates from 3D circumference made in men and women with a Vitus Smart XXL (Human Solutions North America, Cary, NC)^25^ laser scanner. This dataset is referred to as the *Human Solutions* dataset. We had front-, side-, and back-viewpoint color images, height, and body weight for each participant along with their 3D laser scan. The Skinned Multi-Person Linear (SMPL) model was fit to each 3D scan to estimate the shape and pose of the scan^19^. We extracted the ground truth circumferences from the fitted SMPL model at predefined locations (corresponding to hip, waist, chest, thigh, calf and bicep) as shown in Supplementary Figs. 1, 2. Third, we measured the noise in tape measurements compared to MeasureNet using data from a subset of healthy men and women evaluated in the CSD dataset. Each person was measured twice by a trained staff member (staff measurements) and two sets of images were also taken by the staff member (MeasureNet). Each person also measured themselves twice using measuring tape (self-measurements). For staff measurements, each person was measured by two different staff members to ensure minimal correlation between consecutive measurements. We used the difference between two consecutive measurements to analyze the noise distributions of staff-measurements, MeasureNet, and self-measurements.

Lastly, we compared accuracy and repeatability of our approach to the ground truth on a synthetic dataset. We created the dataset by rendering each synthetically generated mesh using different camera parameters (height, depth, focal length) and different body poses placed in front of randomly selected backgrounds. The dataset was generated using synthetic meshes of 100 men and 100 women. This data is referred to as the *Synthetic Dataset*. We considered all of the renderings for a particular mesh to measure repeatability (robustness) of our approach. Repeatability metrics are defined in the Statistical Methods section. Different factors such as background, camera parameters, and body pose changes were present across multiple renderings of the same mesh. A repeatable approach should ideally predict the same output for different renderings of the same mesh. We also use this dataset to evaluate accuracy given all of the renderings and their ground truth.

A flow diagram showing the multiple study human participant evaluations is presented in Supplementary Fig. 3. Consent was obtained for the collection and use of the personal data voluntarily provided by the participants during the study.

### MeasureNet development

**Fig. 3:**
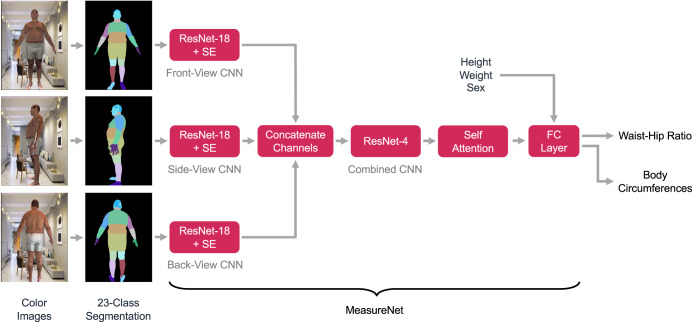
MeasureNet architecture. Overview of the anthropometric body dimension measurement approach. The user first enters their height, weight, and sex into the smartphone application. Voice commands then position the user for capture of front, side, and back color images. The images are then segmented into semantic regions using a segmentation network. The segmentation results are then passed to a second network referred to as MeasureNet that predicts WHR and body circumferences. Each input is passed through a modified Resnet-18 network which is then concatenated and passed through Resnet-4, self-attention block and a fully connected layer (FC layer) before predicting WHR and body circumferences. Resnet-18 is modified to include Squeeze-Excitation blocks (SE). CNN, convoluted neural network. Synthetic images are used to train this model. Real images are used during inference after the model is trained. Color images shown in the figure are synthetically generated.

An overview of our approach for measuring WHR is shown in Fig. 3. The user inputs their height, weight, and sex into their smartphone. Voice commands from the application then guide the person to capture front-, side-, and back-viewpoint color images. The images are then automatically segmented into 23 regions such as the background, upper left leg, lower right arm, and abdomen by a specialized convolutional neural network (CNN) trained to perform semantic image segmentation. Intuitively, this step suppresses irrelevant background features, provides additional spatial context for body parts, and affords important benefits during model training, which we will discuss subsequently. The segmentation result is then passed as input along with the user’s height, weight, and sex into the MeasureNet neural network. MeasureNet then estimates the user’s WHR together with other outputs such as body shape, pose, camera position and orientation, and circumferences such as at the waist, hip, chest, thigh, calf and bicep.

The MeasureNet architecture is built upon a modified Resnet-18 network^26,27^ that “featurizes” each of the three input segmentation images (i.e., transforms each image to a lower-dimensional representation). Features from each view are then concatenated together and fed to a Resnet-4 network and a self-attention network^28^ followed by a fully connected layer to predict body circumferences and WHR as illustrated in Fig. 3.

The following are the key features of the architecture that we found improved accuracy the most:

- Direct prediction of circumferences: Predicting body circumferences directly outperformed first reconstructing the body model (3D SMPL mesh^19^) and then extracting measurements from it.
- Number of input views: Using three views of the user as input improved the accuracy as compared to using one or two views of the user. Tables 2 and 3 shows the improvement in accuracy with increasing number of input views and using direct prediction of circumferences.
- Swish vs. ReLU activations: Resnet typically uses ReLU activations^26^. We found that replacing ReLU with Swish activations^29^ reduced the percentage of “dead” connections (i.e., connections through which gradients do not flow) from around 50% with ReLU to 0% with Swish and improved test accuracy.
- Self-Attention and Squeeze-Excitation for non-local interactions: Including squeeze-excitation blocks^27^ with Resnet branches for cross-channel attention and a self-attention block^28^ after the Resnet-4 block allowed the model to learn non-local interactions (e.g., between bicep and thigh), with further accuracy improvements. Supplementary Note 4 shows the accuracy improvements due to self-attention, squeeze-excitation and Swish activation blocks.
- Sex-specific model: Training separate, sex-specific MeasureNet models further improved accuracy. As we show in Tables 2 and 3, sex-specific models have lower prediction errors compared to sex-neutral models.

MeasureNet predicts multiple outputs, such as body shape, pose, camera, volume, and 3D joints. Predicting multiple outputs in this way (multi-tasking) has been shown to improve accuracy for human-centric computer vision models^30^. Additionally, MeasureNet predicts circumferences and WHR. Some of the outputs (e.g., SMPL shape and pose parameters) are used only to regularize the model during training and are not used during inference^31^. The inputs and outputs to MeasureNet are shown in Fig. S4. Important MeasureNet outputs related to circumferences and WHR are:

- Dense Measurements: MeasureNet predicts 112 circumferences defined densely over the body. Details are presented in Supplementary Note 2. Dense measurements reduce the output domain gap between synthetic and ground truth by finding the circumference ring (out of 112 circumference rings) that minimize the error between tape measurements taken by trained staff and synthetic measurements at a particular ring. The table in Supplementary Note 2 shows that the predicted error at the optimal circumference ring is the lowest and therefore it is well-aligned with the staff measurements.
- WHR Prediction: Our model can predict WHR both indirectly (by taking ratios of waist and hip estimates) and directly (i.e., predicting WHR either through regression or classification). WHR related outputs are shown in Fig. S4. The final WHR prediction is an ensemble result, i.e., we average the individual WHR predictions. As shown in Supplementary Note 5, we found that the ensemble prediction had the lowest repeatability error (most robust) without losing accuracy as compared to individual predictions via regression, classification or taking the ratio of waist and hip.

We include training losses on shape, pose, camera, 3D joints, mesh volume, circumferences and waist-hip ratio (through classification and regression). The losses are defined in Supplementary Note 6. Since we have multiple loss functions, hand-tuning each loss weight is expensive and fragile. Based on Kendall et al.^31^, we used uncertainty-based loss weighting (Eq. 1) where the weight parameter $\left({w}_{i}\right)$ is learned. Uncertainty based loss weighting automatically tunes the relative importance of each loss function $\left({L}_{i}\right)$ based on the inherent difficulty of each task. Supplementary Note 7 shows the improvement in accuracy when using uncertainty-based loss weighting during training.

1 $${\mathscr{L}}=\frac{1}{{w}_{i}}\times {L}_{i}+\log \left(1+{w}_{i}\right)$$

### Realistic synthetic training datasets

**Fig. 4:**
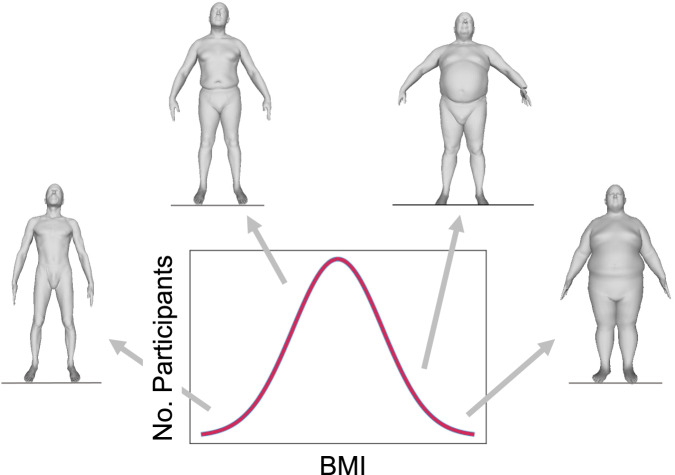
Examples of synthetic body shapes. Examples of diverse synthetically generated body shapes varying in body mass index (BMI).

MeasureNet was trained with synthetic data. Using synthetic data helps avoid expensive, manual data collection and annotation. However, it comes at the cost of synthetic-to-real domain gap, which leads to a drop in accuracy between a model trained with synthetic data but tested on real data. We reduced the domain gap by simulating a realistic image capture process on realistic 3D bodies with lifelike appearance (texture). Examples of synthesized body shapes for different BMI values are shown in Fig. 4.

The SMPL mesh model^19^ is parameterized by shape and pose parameters. To encourage realism in the synthetic dataset and minimize domain gap, it was important to sample only realistic parameters and to match the underlying distribution of body shapes of the target population. Our sampling process was used to generate approximately one million 3D body shapes with ground truth measurements, and consisted of three steps:

- Fit SMPL parameters: Given an initial set of 3500 3D scans (by a laser scanner) as a bootstrapping dataset, we first fitted the SMPL model to all scans^19^ to establish a consistent topology across bodies and to convert each 3D shape into a low-dimensional parametric representation. Due to the high fidelity of this dataset and the variation across participants, we used this dataset as a proxy for the North American demographic distribution of body shapes and poses.
- Cluster samples: We recorded the sex and weight of each scanned subject, and extracted a small set of measurements from the scan, such as height, and hip, waist, chest, thigh, and bicep circumferences. We trained a sex-specific Gaussian Mixture Model (GMM) to categorize the measurements into 4 clusters (we found the optimal number of clusters using Bayesian information criterion).
- Sample the clusters using importance sampling: Finally, we used importance sampling to match the likelihood of sampling a scan to match the distribution across all clusters. This allowed us to create a large synthetic dataset of shape and pose parameters whose underlying distribution matched the diversity of the North American population. As an additional check, we found that our dataset created using the above method closely matched the distribution of the NHANES dataset (https://www.cdc.gov/nchs/nhanes/about_nhanes.htm). NHANES was collected by the Center for Disease Control and Prevention between the years 1999 and 2020 and consists of the demographics, body composition and medical conditions of about 100,000 unique participants from North American population.

**Fig. 5:**
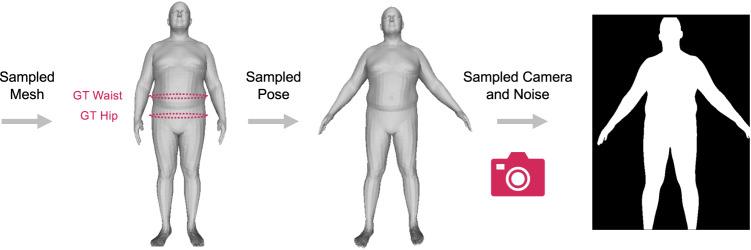
Realistic shape sampling. Example of realistic sampling of body shape, pose, and camera simulating the image capture process.

We simulated a realistic capture process by sampling across the range of all possible camera orientations (in the range of −15 to +15 degrees around each axis) that yielded valid renderings of the user in the input image. Valid renderings are images in which body shapes are visible from at least the top of the head to the knees. This ensures that the sampled camera parameters match the realistic distribution of camera parameters observed for real users. An example of realistic sampling of shape, pose, and camera are shown in Fig. 5.

Once body shape, body pose, and camera orientation were sampled, we transferred the texture from a real person onto the 3D mesh, placed it in front of a randomly selected background image (of an indoor scene) and rendered a realistic color rendering given the camera pose. The textured and realistic color rendering was then segmented using the segmentation network that was used as an input to train MeasureNet. The ground truth targets used to train MeasureNet were extracted from sampled synthetic mesh. Transferring the texture from a real person allowed us to generate diverse and realistic samples and had two main advantages. First, we transferred the texture from a real person which avoided manually generating realistic and diverse textures. Through this method, we generated a texture library of forty thousand samples using trial users (different from test-time users). Second, since we segmented the color images using a trained segmentation model, we did not have to include additional segmentation noise augmentation^30^ during training. This is in contrast to the existing methods^21,30^ that add segmentation noise to the synthetic image in order to simulate the noisy segmentation output during test-time. We used the segmented image as input to MeasureNet instead of a textured color image to force MeasureNet to not use any lighting or background-related information from the synthetic training data which can have different distributions during training and testing. In Supplementary Note 8, we show that training a model with textured color image generalizes poorly when tested on real examples as compared to segmented images. Intuitively, we believe this is the case because synthetic textured color images lack realism on their own, but generate realistic segmentation results when passed through a semantic segmentation model.

**Fig. 6:**
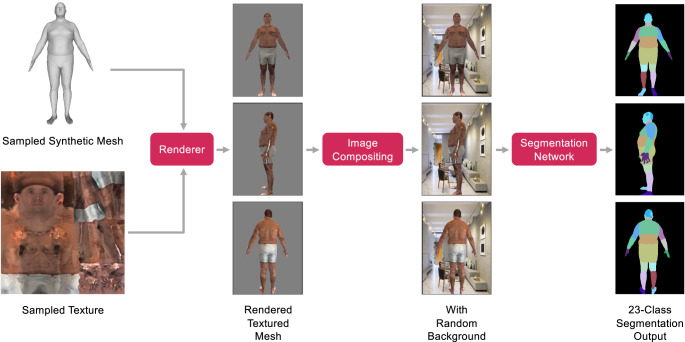
Realistic synthetic dataset generation. Generation of realistic color mesh renderings by transferring texture from a real person (synthetic in this example). The renderings when segmented using a fixed network are used as input to train MeasureNet. The ground truth targets used to train MeasureNet are extracted from sampled synthetic mesh.

**Fig. 7:**
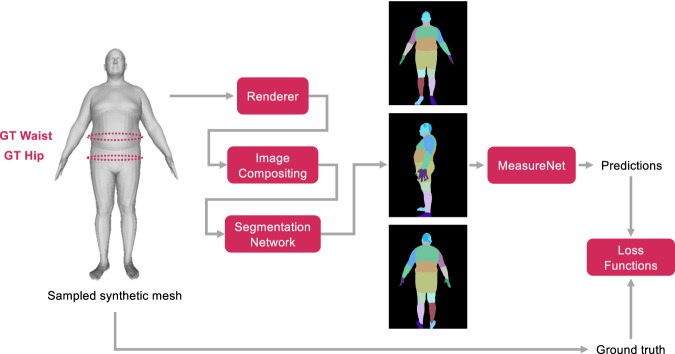
MeasureNet training. Training of MeasureNet model using realistic synthetic data. Given a sampled synthetic mesh, realistic synthetic images are generated that are segmented. The segmented images are used as input to MeasureNet and corresponding predictions are compared against the ground truth extracted from synthetic mesh.

Overall, the texture transfer process consisted of two steps. First, we created a texture library by extracting textures from real images using our participant pipeline. We extracted around forty thousand texture images from trial users. Second, given the texture images, we rendered a randomly sampled synthetic mesh using a random texture image, rendered it on a random background, and passed it through the segmentation. The process of realistic textured rendering by transferring the texture from a real person (synthetic in this case) is shown in Fig. 6. The renderings when segmented (using fixed segmentation network) were used as input to train MeasureNet. The end-to-end training process for MeasureNet is shown in Fig. 7. The ground truth targets used to train MeasureNet are extracted from sampled synthetic mesh.

### Statistical methods

The accuracy of MeasureNet and self-measurements were compared to trained staff-measured ground truth estimates in the CSD using mean absolute error (MAE; Eq. 2) and mean absolute percentage error (MAPE; Eq. 3) metrics. MAE calculates the average relative error of MeasureNet’s prediction or self-measurements with respect to the ground truth tape measurements. MAPE is similar to MAE but calculates mean relative percentage error.

2 $${MAE}=\frac{{\sum }_{i=1}^{n}\left|{G}_{i}-{P}_{i}\right|}{n}$$

3 $${MAPE}=\frac{100 \% }{n}\mathop{\sum }\limits_{i=1}^{n}\left|\frac{{G}_{i}-{P}_{i}}{{P}_{i}}\right|$$

*G*_*i*_ is the ground truth, *P*_*i*_ is the prediction, and *n* is the number of users. MAE was also used for comparing MeasureNet to other state-of-the-art approaches for estimating circumferences and WHR.

The same procedures were used for evaluating noise in staff measurements, MeasureNet predictions, and self-measurements. This analysis is used to compare the measurement noise of flexible tape-based measurements (staff and self) and MeasureNet’s predictions. Noise was estimated by plotting histograms of the between-measurement or prediction differences (meas_1_ and meas_2_). Biases in differences were removed before plotting the histograms by including the Δs in both directions: meas_1_ – meas_2_ and meas_2_ – meas_1_. We also fit Gaussian curves on the resulting histograms to estimate the noise standard deviations.

Repeatability was computed on the synthetic dataset and measured as the mean and 90^th^ percentile (P90) of absolute differences. The repeatability metric was computed using the following steps: (1) The mean estimate (*µ*) was computed for each session consisting of renderings where the same synthetic mesh is rendered given different camera parameters, different body poses, and placed in front of a random background (2) for each scan we computed the absolute difference to the mean of that session (|*pred*−*µ*|), and (3) we computed the mean and P90 of absolute differences across all scans.

### Ethics review

The participant data evaluated in this study is approved by PBRC Institutional Review Boards (clinicaltrials.gov identifier: NCT04854421). The reported investigation extends the analyses to anthropomorphic data (waist and hip circumference measurements), and reflects a secondary analysis of data collected by Amazon vendors in commercial settings. All participants signed consents in these no-risk studies that granted full permission to use their anonymized data. The investigators will share the data in this study with outside investigators upon request to and approval by the lead author.

### Reporting summary

Further information on research design is available in the Nature Research Reporting Summary linked to this article.

### Supplementary information

Supplementary Material

Reporting Summary

## Data Availability

The data that supports the findings of this study is available from the corresponding author upon reasonable request and approval of Amazon Ltd.

## Code availability

The code that supports the findings of this study is available from the corresponding author upon reasonable request and approval of Amazon Ltd. Custom scripts for data processing were developed in MATLAB 2020a and statistical analyses were performed in Python version 3.7.

## References

[1] Vague J (1947). Sexual differentiation; Factor determining forms of obesity. Presse Med..

[2] Krotkiewski M, Bjorntorp P, Sjostrom L, Smith U (1983). Impact of obesity on metabolism in men and women. Importance of regional adipose tissue distribution. J. Clin. Investig..

[3] Larsson B (1984). Abdominal adipose tissue distribution, obesity, and risk of cardiovascular disease and death: 13 year follow up of participants in the study of men born in 1913. Br. Med. J..

[4] Bjorntorp P (1987). Fat cell distribution and metabolism. Ann. N. Y Acad. Sci..

[5] World Health Organization (WHO). *Waist circumference and waist-hip ratio: report of a WHO expert consultation* (World Health Organization, 2011).

[6] Cameron AJ, Magliano DJ, Soderberg S (2013). A systematic review of the impact of including both waist and hip circumference in risk models for cardiovascular diseases, diabetes and mortality. Obes. Rev..

[7] Cerhan JR (2014). A pooled analysis of waist circumference and mortality in 650,000 adults. Mayo Clin. Proc..

[8] Jacobs EJ (2010). Waist circumference and all-cause mortality in a large US cohort. Arch. Intern Med..

[9] Ross R (2020). Waist circumference as a vital sign in clinical practice: a Consensus Statement from the IAS and ICCR Working Group on Visceral Obesity. Nat. Rev. Endocrinol..

[10] Seidell JC (2010). Waist circumference and waist/hip ratio in relation to all-cause mortality, cancer and sleep apnea. Eur. J. Clin. Nutr..

[11] Criminisi A, Sorek N, Heymsfield SB (2022). Normalized sensitivity of multi-dimensional body composition biomarkers for risk change prediction. Sci. Rep..

[12] Sebo P, Beer-Borst S, Haller DM, Bovier PA (2008). Reliability of doctors’ anthropometric measurements to detect obesity. Prev. Med..

[13] Sebo P, Haller D, Pechere-Bertschi A, Bovier P, Herrmann F (2015). Accuracy of doctors’ anthropometric measurements in general practice. Swiss Med. Wkly.

[14] Sebo P, Herrmann FR, Haller DM (2017). Accuracy of anthropometric measurements by general practitioners in overweight and obese patients. BMC Obes..

[15] Majmudar MD (2022). Smartphone camera based assessment of adiposity: a validation study. NPJ Digit Med..

[16] Smith B (2022). Anthropometric evaluation of a 3D scanning mobile application. Obesity.

[17] Sengupta A., Budvytis I., Cipolla R. Hierarchical Kinematic Probability Distributions for 3D Human Shape and Pose Estimation from Images in the Wild. *2021 International Conference on Computer Vision*, (2021). pp. 11199–11209, 10.1109/ICCV48922.2021.01103

[18] Heymsfield SB (2018). Digital anthropometry: a critical review. Eur. J. Clin. Nutr..

[19] Loper M, Mahmood N, Romero J, Pons-Moll G, Black MJ (2015). SMPL: A Skinned Multi-Person Linear Model. ACM Trans. Graph..

[20] Kolotouros N., Pavlakos G., Black M. J., Daniilidis K. Learning to Reconstruct 3D Human Pose and Shape via Model-Fitting in the Loop. *2019 IEEE/CVF International Conference on Computer Vision (ICCV)*, 2252–2261 (2019). (IEEE, 2019)

[21] Sengupta A., Budvytis I., Cipolla R. Synthetic training for accurate 3D human pose and shape estimation in the wild. *2020 British Machine Vision Conference (BMVC)*, (2020). (British Machine Vision Association, 2020)

[22] Bogo F., et al Keep It SMPL: Automatic Estimation of 3D Human Pose and Shape from a Single Image. In: *Computer Vision – ECCV 2016* (eds Leibe B., Matas J., Sebe N., Welling M.). (Springer International Publishing, 2016).

[23] Chen L, Peng S, Zhou X (2021). Towards efficient and photorealistic 3D human reconstruction: a brief survey. Vis. Inform..

[24] Kanazawa A., Black M. J., Jacobs D. W., Malik J. End-to-End Recovery of Human Shape and Pose. *2018 IEEE/CVF Conference on Computer Vision and Pattern Recognition*, 7122–7131 (2018). (IEEE, 2018)

[25] Maurer M. VITUS 3D Body Scanner. Asian Workshop on 3D Body Scanning Technologies: http://www.3dbody.tech/A2012/programasia.html (2012).

[26] He K., Zhang X., Ren S., Sun J. Deep residual learning for image recognition. *2016 IEEE Conference on Computer Vision and Pattern Recognition (CVPR)*, 770–778 (2016). (IEEE, 2016)

[27] Hu J, Shen L, Albanie S, Sun G, Wu E (2020). Squeeze-and-excitation networks. IEEE Trans. Pattern Anal. Mach. Intell..

[28] Wang X., Girshick R. B., Gupta A. K., He K. Non-local neural networks. *2018 IEEE/CVF Conference on Computer Vision and Pattern Recognition*, 7794–7803 (2018). (IEEE, 2018)

[29] Ramachandran P., Zoph B., Le Q. V. Searching for Activation Functions. *ArXiv***abs/1710.05941**, (2018).

[30] Smith B. M., Chari V., Agrawal A., Rehg J. M., Sever R. Towards accurate 3D human body reconstruction from silhouettes. *2019 International Conference on 3D Vision (3DV)*, 279–288 (2019). (IEEE, 2019)

[31] Kendall A., Gal Y., Cipolla R. Multi-task learning using uncertainty to weigh losses for scene geometry and semantics. *Proceedings of the 2018 IEEE Conference on Computer Vision and Pattern Recognition (CVPR)*, 7482–7491 (2018). (IEEE, 2018)

