# Supplementary material for: Development and validation of an accurate smartphone application for measuring waist-to-hip circumference ratio
Source: NPJ Digit Med. 2023 Sep 11;6:168. doi: 10.1038/s41746-023-00909-5 (PMC10495406; doi:10.1038/s41746-023-00909-5)
Supplement: Supplementary file 1 — Supplementary Material [file 41746_2023_909_MOESM1_ESM.pdf]

SUPPLEMENTARY MATERIAL

Development and Validation of an Accurate Smartphone Application for Measuring the Waist to Hip Circumference Ratio

Siddharth Choudhary[1*], Ganesh Iyer[1], Brandon M. Smith[1], Jinjin Li[1], Mark Sippel[1], Antonio Criminisi[1], Steven B. Heymsfield[1,2]

[1]Amazon Inc., Seattle, USA; [2]Pennington Biomedical Research Center, Louisiana State University System, Baton Rouge, USA.

Address Correspondence to:
Siddharth Choudhary, PhD
ORCID ID: 0000-0001-5329-1858
Amazon, Inc.
2121 7th Avenue
Seattle, WA USA 98121
E-mail: sidchoud@amazon.com

1. **Circumference Measurement Protocol**
2. **Synthetic Ground Truth Measurement**
3. **Study Flow Diagram**
4. **Additional ablation results with ReLU activations, Self-attention (SA) and Squeeze-Excitation (SE) blocks.**
5. **Predicting Waist-Hip ratio through Regression vs. Classification**
6. **MeasureNet Training Details**
7. **Training MeasureNet with uncertainty-based loss weighting**
8. **Training MeasureNet using synthetic textured color images**
9. **Participant Characteristics**
10. **Qualitative Comparisons Between Predicted and Ground Truth Meshes**
11. **Noise Distributions**

1. **Circumference Measurement Protocol**

Neck

- Participant standing in relaxed posture with head in neutral position.
- Locate the upper end of the neck where the neck meets the jaw
- Locate the base of the neck, just above the shoulder.
- Measure is taken at midway point between two above locations. Measuring tape is perpendicular to neck axis.

Chest For Men / Bust for Women

- Participant standing in relaxed A-pose (best effort for self-measure).
- Measure is taken at area of maximal circumference of upper torso region.
- Take care to ensure that tape measure does not have excess distance across sides and back.

Arm

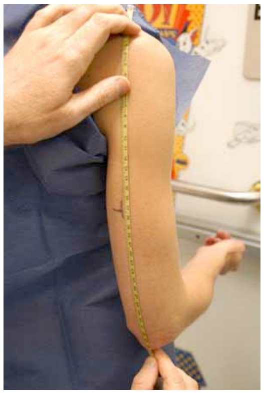

- Measures to be taken of the right arms.
- Participant standing in relaxed posture with arm to be measured at a 90 degree angle with the palm facing up.
- Follow the participant's spine of the right scapula until it makes a sharp V at the shoulder. Note the shoulder at this location.
- Measure the arm from this point to the tip of the elbow and carefully make sure the measuring tape is in the center of the posterior surface of the arm. Note the midpoint of the arm.
- Ask the participant to stand with both arms hanging loosely at their side and weight evenly distributed on both feet. The participant should not flex or tighten any muscles.
- Measure the circumference at the previous noted midpoint of the arm. The tape should be perpendicular to the long axis of the upper arm.

Waist

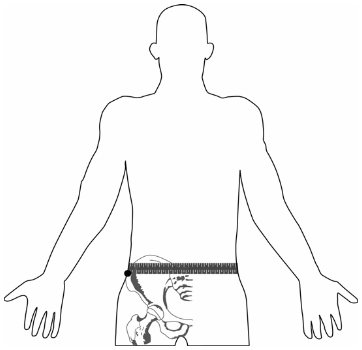

- Have participant stand with weight evenly distributed on both feet in relaxed A-pose.
- Locate the right ilium of the pelvis and draw a line just above the lateral border of the right ilium.
- Place the measuring tape around the participant making sure it is snug but not compressing skin. The measuring tape should be completely parallel to the ground.

Hip

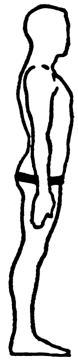

- Have participant stand with weight evenly distributed on both feet.
- The measuring tape is placed around the buttocks at the point of maximal circumference. The sides of the tape should be checked to ensure the tape is horizontal. The tape should be held snug but not tight.
- The value should be read at the right side of the participant.

Thigh

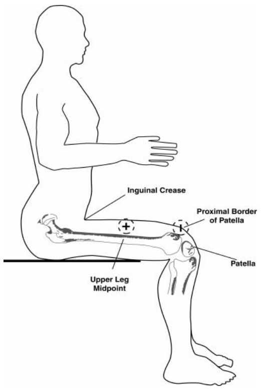

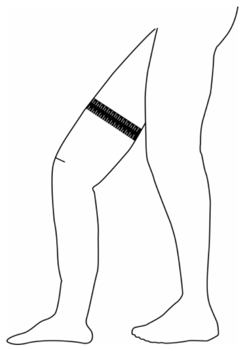

- Measures to be taken of the right thigh.
- Locate midpoint of thigh by placing zero end of measuring tape at the inguinal crease. Folds of fat tissue may have to be lifted to reach the crease. Extend tape down towards the line created at the proximal end of the patella. To be sure you have the zero end of the tape at the inguinal crease, place your thumb at the zero end of the tape and instruct the participant to slightly lift the thigh. A tightening of muscle tendon should be felt. Note the midpoint.
- Measure from the patella to 1/3 the length. Note this point.
- After noting the midpoint, have the participant stand with most of his or her weight on the non-measured leg. The measured leg should be slightly bent and forward. The participant may use an object (such as a table) for balance.
- Place the measuring tape around the mid-thigh as noted. The tape should be perpendicular to the long axis of the thigh.

Calf

- Measures to be taken of the right calf.
- Locate the point of maximal circumference between the knee and ankle.
- Have the participant stand in a relaxed A-pose.
- Wrap the measuring tape around the lower leg at the noted point of maximal circumference.
- The tape should be perpendicular to the long axis of the lower leg.

Forearm

- Measures to be taken of the right forearm.
- Have the participant stand with arm hanging loosely at their side and weight evenly distributed on both feet. The participant should not flex or tighten any muscles.

- Locate the point of maximal circumference between the elbow and wrist.
- Wrap the measuring tape around the forearm at the noted point of maximal circumference.
- The tape should be perpendicular to the long axis of the forearm.

2. Synthetic Ground Truth Measurement

The method to estimate ground truth measurement rings on the synthetic mesh that align with the tape measured ground truth was as follows. First, 112 dense circumferences were defined over the body as shown in **Figure 1**. The dense circumferences (shown in red) consisted of the following:

1. 20 torso circumferences
2. 6 upper left arm circumferences
3. 8 lower left arm circumferences (including elbow)
4. 6 upper right arm circumferences
5. 8 lower right arm circumferences
6. 10 upper left leg circumferences
7. 11 lower left leg circumferences
8. 10 upper right leg circumferences
9. 11 lower right leg circumferences

Each circumference was found by intersecting a plane with the SMPL mesh at specific predefined intersection points and finding the circumference at that intersection. These points and the corresponding intersection planes were found as follows:

- We first find 23 3D joint locations over the mesh using the SMPL parametric joint regressor. We also define skeletal axes that connect these 3D joints (as shown by green lines in **Figure 2**).
- We sub-divide each axis based on the average body part length to find the intersection points. For e.g., upper left/right arm circumferences consist of 8 locations where intersections are found between the left shoulder joint and left elbow joint locations respectively. To make sure that the plane does not self-intersect at multiple points on the mesh, we segment the part specific vertices and faces before finding the intersection.

- Lastly, we refine the orientation of the plane before intersection. We re-estimate the orientation such that intersecting plane is perpendicular to the surface of the mesh for the specified body part. This ensures a realistic and robust circumference estimation method that can generalize across the variation of body shapes.

Next, we identify the correct part specific circumference locations on the SMPL mesh which aligns with tape measurements. We find these locations using a data-driven approach using a dataset containing 3D scans and tape measurements. For each part, we collect dense measurements using the above protocol and compare the circumference with the mean of two tape measurements taken by a trained staff member. The location index that minimizes the MAE (Eq. 1) for each part is considered as ground truth on SMPL meshes. This reduces the output domain gap between the ground truth circumference definition on synthetic meshes which used to train MeasureNet and tape measurements taken by a trained staff member. Ground truth circumference rings which align with tape measurements are shown in **Figure 2** for the segmented regions.

Supplementary Figure 1. Dense circumference locations. The MeasureNet algorithm predicts 112 circumferences defined densely over the body. Dense circumferences are shown in red and are uniformly sampled over the body.

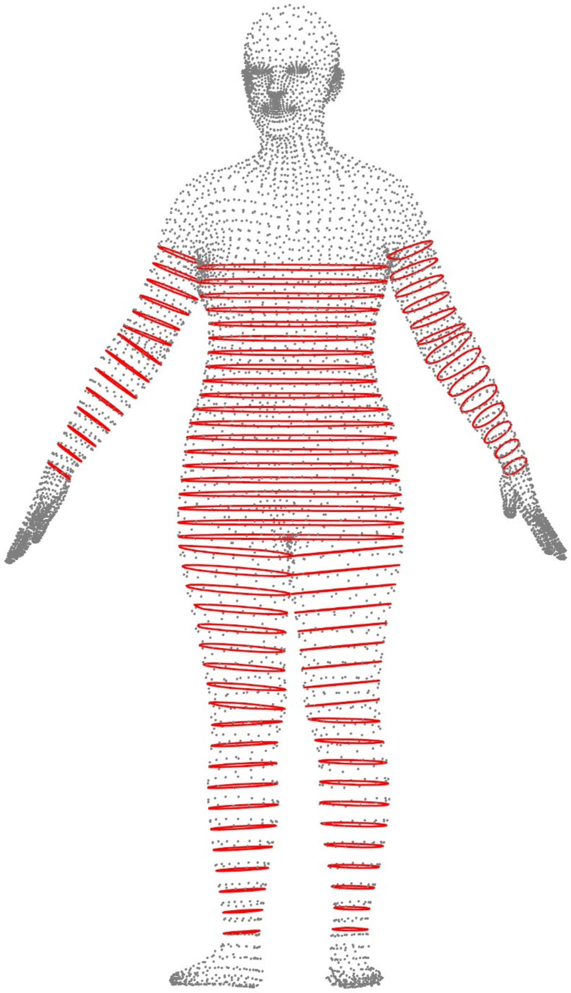

Supplementary Figure 2. Tape measurements aligned synthetic circumference rings. Segmented vertices and faces are shown in color. 3D joint locations are shown in pink. Ground truth circumference rings which align with tape measurements are shown in white for the segmented regions.

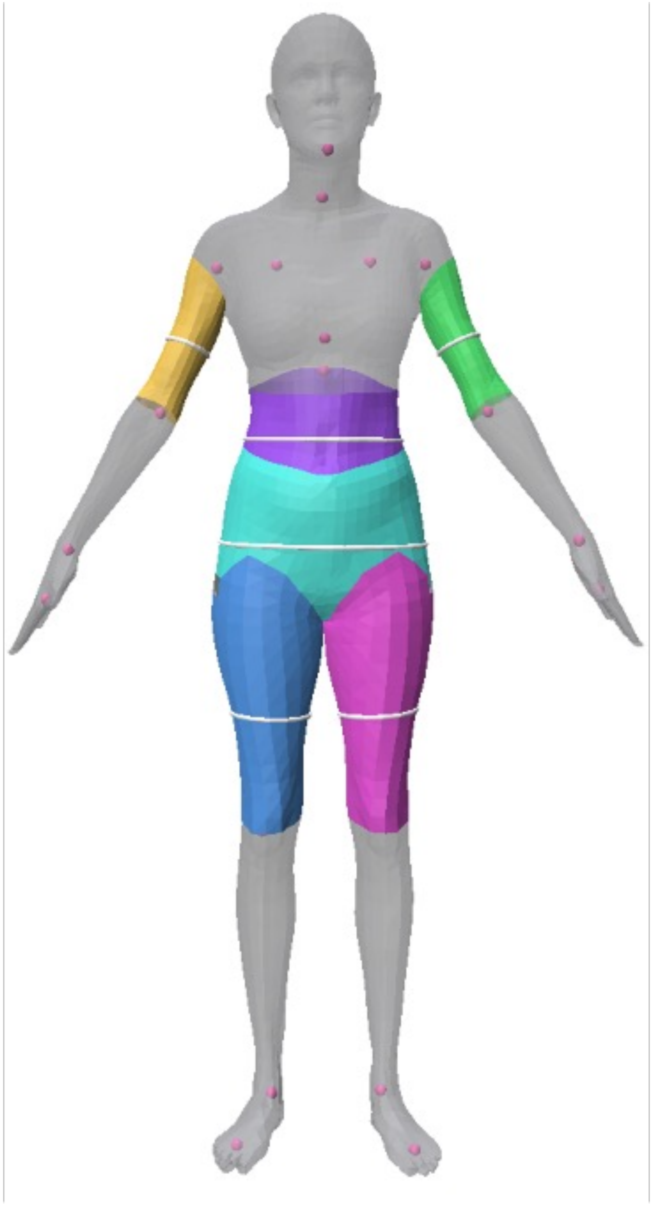

The table below compares the mean absolute error in hip and waist as compared to staff tape measurement (from CSD dataset) given the MeasureNet predictions at and around optimal ring location. Accuracy is relative to ground truth staff measurements on Circumference Study Dataset (CSD). We compare the error between optimal ring index and two rings around the optimal index. It shows that the predicted error at optimal ring index is the lowest when compared to ring indices around it.

**Method**	**Accuracy on CSD**	
	Hip MAE (mm)	Waist MAE (mm)
**Men**		
Optimal Ring Index - 2	26.93	22.23
Optimal Ring Index - 1	22.28	**20.89**
Optimal Ring Index	**20.68**	21.72
Optimal Ring Index + 1	21.18	24.66
Optimal Ring Index + 2	23.45	31.42
**Women**		
Optimal Ring Index - 2	27.56	28.24
Optimal Ring Index - 1	23.13	25.28
Optimal Ring Index	**20.39**	**24.79**
Optimal Ring Index + 1	21.47	25.43
Optimal Ring Index + 2	27.08	26.84

3. Study Flow Diagram

Supplementary Figure 3. Disposition of Study Participants.

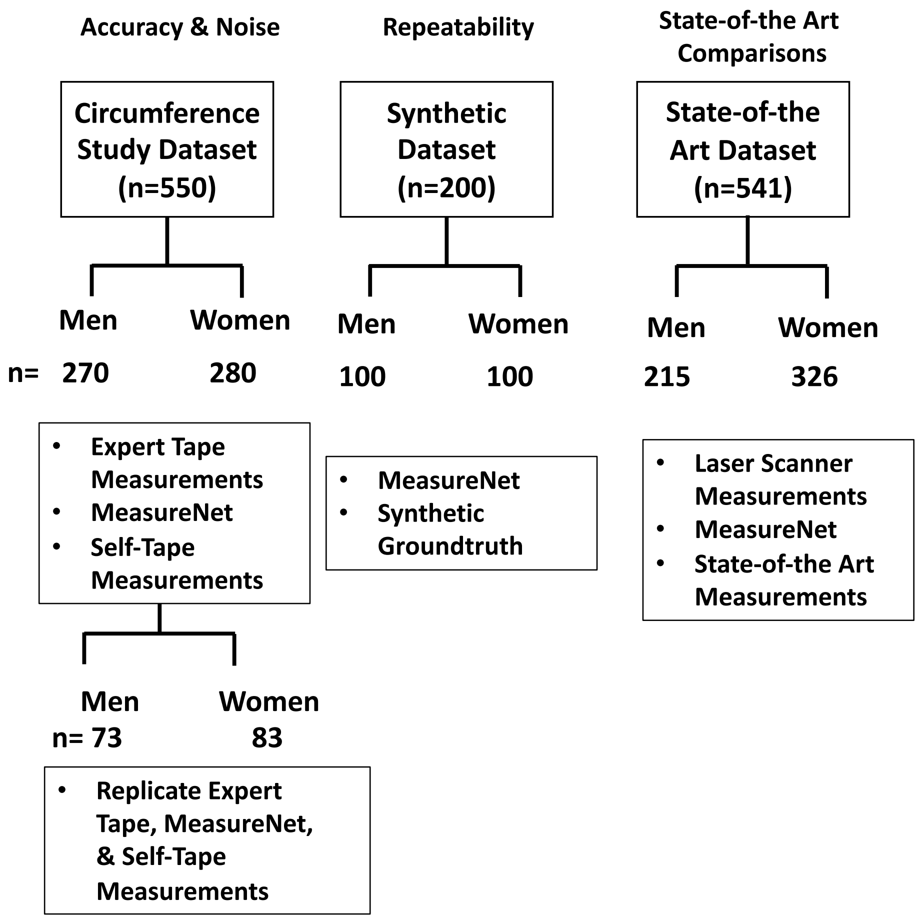

4. **Additional ablation results with ReLU activations, Self-attention (SA) and Squeeze-Excitation (SE) blocks.**

The table below compares MeasureNet models trained with ReLU or Swish activation, with or without Self-attention (SA) and Squeeze-Excitation (SE) blocks. The models were tested on Human Solutions dataset. Lower is better.

	**Hip (mm)**	**Waist (mm)**	**Chest (mm)**	**Thigh (mm)**	**Calf (mm)**	**Bicep (mm)**
**Men**						
Using ReLU + No SA and SE blocks	15.93	18.49	19.31	12.48	10.52	9.30
Using Swish + No SA and SE blocks	15.86	16.56	17.49	12.47	9.86	8.72
Using Swish + Using SA and SE blocks	**14.38**	**16.38**	**15.90**	**11.69**	**9.67**	**7.95**
**Women**						
Using ReLU + No SA and SE blocks	31.82	28.84	24.68	12.74	9.30	12.80
Using Swish + No SA and SE blocks	24.74	26.59	24.96	16.37	11.40	10.22
Using Swish + Using SA and SE blocks	**14.43**	**21.67**	**20.07**	**11.07**	**7.14**	**8.16**

5. Predicting Waist-Hip ratio through Regression vs. Classification

WHR can be predicted indirectly (by taking ratios of waist and hip estimates) and directly (either through regression or classification). The table below compares the accuracy using different methods. We use the average of WHR predictions through classifications, regression and ratio of predicted waist and hip circumferences as the final WHR prediction. We found that the average prediction was the most robust without losing accuracy as compared to predictions via regression, classification or taking the ratio of waist and hip. Accuracy is relative to ground truth staff measurements on Circumference Study Dataset (CSD).

**Sex**	**Method**	**Accuracy on CSD**		**Repeatability on Synthetic Data**	
		MAE	P90	MAE	P90
**Men**	Direct Regression	0.0122	0.042	0.0059	0.0127
	Soft Classification (Expectation)	0.0122	0.0408	0.0057	0.0123
	Soft Classification (Maximum)	0.0123	0.0445	0.0065	0.0139
	Soft Classification (Gaussian Fit)	0.0122	0.0404	0.0057	0.0123
	Ratio of Waist and Hip	0.012	0.0408	0.0058	0.0126
	Average of above methods	0.0122	0.0415	0.0057	0.0123
**Women**	Direct Regression	0.0173	0.0501	0.0061	0.0125
	Soft Classification (Expectation)	0.0169	0.0484	0.0058	0.0122
	Soft Classification (Maximum)	0.0171	0.0508	0.0065	0.0135
	Soft Classification (Gaussian Fit)	0.0169	0.0488	0.0057	0.012
	Ratio of Waist and Hip	0.0168	0.0477	0.006	0.0124
	Average of above methods	0.0169	0.0483	0.0058	0.012

†

6. MeasureNet Training Details

Outputs: MeasureNet predicts multiple outputs, such as body shape, pose, camera, volume, 3D joints, body circumferences and waist-hip ratio (WHR). WHR is predicted through direct regression, classification and indirect prediction via ratio of waist and hip circumferences. **Supplementary Figure 4** shows all the outputs. We explain each of the outputs below:

1. Body Shape: Body shape and pose are represented using SMPL parameters. SMPL (Skinned Multi-Person Linear) model is a realistic data-driven model of 3D human shape and pose. Body shape parameters consists of 300 scalar values, each of which could be interpreted as amount of expansion/shrink of a human subject along some direction such as taller or shorter.
2. Body Pose: It is represented using 24 x 3 scalar values containing the relative rotations for each skeleton joint with respect to its parent joint in a kinematic chain. Each rotation for 24 joint landmarks is encoded as a 3D vector in axis-angle rotation representation.
3. Body Mesh: Given the body shape and pose, we can use SMPL parametric model to generate the mesh. Given the mesh, we can extract the vertices. Vertices are represented using 6890 x 3 scalar values containing 3D location for 6890 vertices. Mesh consists of 13376 faces. All body meshes share the same pre-defined topology on 6890 vertices.
4. Body Volume: Volume for a mesh is represented using a single scalar value. Given the mesh, we can extract body volume by computing the signed volume of a tetrahedron.
5. 3D Joints: Given the body mesh, each 3D joint location is estimated as a linear combination of surrounding vertices. The linear combination is represented by a joint regression matrix that defines a sparse set of vertex weight for each joint. 3D joints are represented using 24 x 3 corresponding to X, Y, Z location for 24 joint landmarks.
6. Camera Parameters: Camera consists of rotation and translation parameters. Rotation is represented using 3 x 3 matrix of scalar values that represents a

rotation around the three axes of a coordinate system. Translation is specified using 3 x 1 vector which represents the distance of camera from the user.

7. Body Circumferences and WHR: Circumferences and WHR are represented using a scalar value. Circumferences are extracted from the mesh at pre-defined locations for each body part. Details are presented in **Supplementary Note 2.**

Training Loss

MeasureNet model is trained with the following losses corresponding to each output:

1. Vertex Loss: Minimizes the mean L2 error between predicted and ground truth shape vertices in A-pose.

$$\mathcal{L}_{\text{vertex}} = \frac{1}{V}\sum_{j}^{V} \|v_j^{\text{pred}} - v_j^{\text{true}}\|^2 \quad (1)$$

2. Volume loss: Minimizes the L2 error between predicted and ground truth mesh volume.

$$\mathcal{L}_{\text{volume}} = \|\text{volume}^{\text{pred}} - \text{volume}^{\text{true}}\|^2 \quad (2)$$

3. Pose loss: Minimizes the mean L2 error between the predicted and ground truth joint angles.

$$\mathcal{L}_{\text{pose}} = \frac{1}{N_{\text{joints}}}\sum_{j}^{N_{\text{joints}}} \|\text{pose}_j^{\text{pred}} - \text{pose}_j^{\text{true}}\|^2 \quad (3)$$

4. 3D Joints loss: Minimizes the mean L2 error between the predicted and ground truth joint locations.

$$\mathcal{L}_{\text{joints}} = \frac{1}{N_{\text{joints}}}\sum_{j}^{N_{\text{joints}}} \|\text{joints}_j^{\text{pred}} - \text{joints}_j^{\text{true}}\|^2 \quad (4)$$

5. Camera loss: Minimizes the L2 error between predicted and ground truth camera translations and rotations.

$$\mathcal{L}_{\text{camera}} = \|\text{camera}^{\text{pred}} - \text{camera}^{\text{true}}\|^2 \quad (5)$$

6. Circumference loss: Minimizes the mean L1 error between the predicted and ground truth circumferences over all the dense circumferences.

$$\mathcal{L}_{\text{circ}} = \frac{1}{N_{\text{parts}}} \sum_{j}^{N_{\text{parts}}} \|\text{circ}_j^{\text{pred}} - \text{circ}_j^{\text{true}}\| \qquad (6)$$

7. WHR regression loss: Minimizes the L1 error between the predicted and ground truth WHR value.

$$\mathcal{L}_{\text{whr}} = \|\text{WHR}^{\text{pred}} - \text{WHR}^{\text{true}}\| \qquad (7)$$

8. WHR classification loss: We found that casting the WHR prediction problem as a classification task yielded higher accuracy than using the more intuitive regression formulation. The different methods are compared in **Supplementary Note 5**. To convert the regression problem into a classification one, we divided the full WHR range (0.5-1.5) into 100 bins and smoothed the output class probabilities. The WHR value when then computed as the expectation of the estimated class distribution. The classification approach also enabled us to estimate the output uncertainty which could then be used for out-of-distribution detection.

Implementation Details

The training and evaluation code was implemented in Python using Pytorch framework. We use 640 x 360 as the input resolution for the image. Synthetic data was generated offline and was fixed during training. The model was trained for 400 epochs using Adam optimizer with a learning rate of 1e-4.

Supplementary Figure 4. Inputs and outputs of the MeasureNet model. Input are three semantically segmented images of the user from front, side and back view along with metadata information including height, weight, and sex. MeasureNet predicts multiple outputs, such as body shape, pose, camera, volume, 3D joints, circumferences, and waist-hip ratio (WHR).

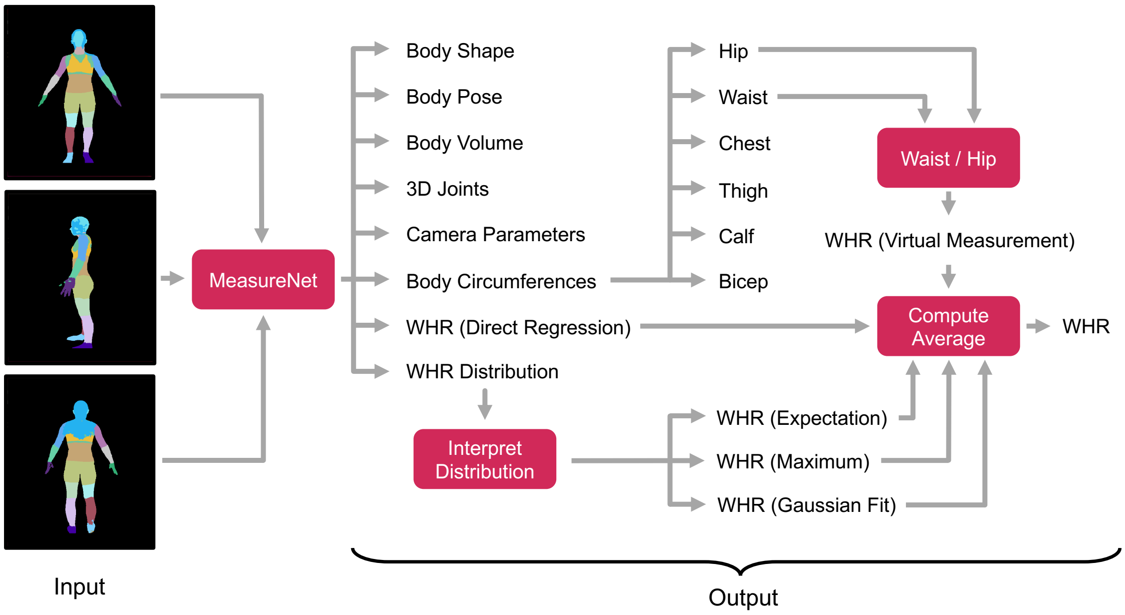

7. Training MeasureNet with uncertainty-based loss weighting[4]

Since we have multiple loss functions corresponding to each output in MeasureNet, hand-tuning each loss weight is expensive and fragile. Based on Kendall et al.[4], we used uncertainty-based loss weighting where the weight parameter is learned. The table below shows the improvement in accuracy using uncertainty-based loss weighting when tested on Human Solutions dataset. Lower is better.

	Hip (mm)	Waist (mm)	Chest (mm)	Thigh (mm)	Calf (mm)	Bicep (mm)
**Men**						
Without uncertainty-based loss weighting	15.50	19.24	18.41	12.18	10.24	8.69
With uncertainty-based loss weighting[4]	**14.38**	**16.38**	**15.90**	**11.69**	**9.67**	**7.95**
**Women**						
Without uncertainty-based loss weighting	24.48	24.25	22.91	11.21	8.06	9.66
With uncertainty-based loss weighting[4]	**14.43**	**21.67**	**20.07**	**11.07**	**7.14**	**8.16**

8. Training MeasureNet using synthetic textured color images

The table shows that training a model with synthetic textured color images generalizes poorly when tested on real examples from Human Solutions dataset as compared to using 23-class segmented images as input. These models were trained using a subset of synthetic dataset. The numbers in the table show the circumference prediction error when using color image or 23-class segmented image as an input. Lower is better.

Input Method	Hip (mm)	Waist (mm)	Chest (mm)	Thigh (mm)	Calf (mm)	Bicep (mm)
**Men**						
Color Image	16.92	**17.40**	19.70	11.78	13.68	**8.80**
23-class Segmentation Image	**16.06**	17.90	**17.30**	**10.73**	**10.41**	8.92
**Women**						
Color Image	29.53	24.40	**22.39**	12.22	9.71	10.24
23-class Segmentation Image	**22.44**	**21.76**	23.33	**9.97**	**8.26**	**9.82**

9. Participant Characteristics.

A. Demographic and anthropometric characteristics for Circumference Study Dataset. BMI, body mass index. Data are X± 3SD.

		All	Men	Women
**Total Number**		550	270	280
	White	200 (36.3%)	97 (35.9%)	103 (36.8%)
	Black	80 (14.5%)	40 (14.8%)	40 (14.3%)
**Ethnicity**	Asian	98 (17.8%)	57 (21.1%)	41 (14.6%)
	Hispanic	147 (26.7%)	59 (21.8%)	88 (31.4%)
	American Indian	2 (0.4%)	1 (0.4%)	1 (0.3%)
	Others	23 (4.2%)	16 (5.9%)	7 (2.5%)
**Height (cm)**		169.79 ± 31.35	176.74 ± 24.12	163.09 ± 23.40
**Weight (kg)**		78.48 ± 54.33	86.40 ± 50.73	70.85 ± 47.46
**Body Mass Index (kg/m2)**		27.16 ± 17.16	27.71 ± 16.95	26.64 ± 17.22
**Waist Circumference (cm)**		90.35 ± 39.00	91.91 ± 38.04	88.85 ± 39.45
**Hip Circumference (cm)**		102.72 ± 31.53	102.23 ± 28.68	103.20 ± 34.05
**Waist-to-hip ratio**		0.877 ± 0.201	0.896 ± 0.186	0.859 ± 0.201

B. Demographic and anthropometric characteristics for Human Solutions dataset used in comparison with state-of-the-art approaches. Data are X± 3SD.

		All	Men	Women
**Total Number**		541	215	326
	White	368 (68.0%)	132 (61.4%)	236 (72.4%)
	Black	120 (22.2%)	64 (29.7%)	56 (17.2%)
**Ethnicity**	Asian	6 (1.1%)	2 (0.9%)	4 (1.2%)
	Hispanic	16 (2.9%)	6 (2.8%)	10 (3.0%)
	American Indian	3 (0.5%)	1 (0.5%)	2 (0.6%)
	Others	28 (5.1%)	10 (4.6%)	18 (5.5%)
**Height (cm)**		170.12 ± 27.66	177.85 ± 21.93	165.03 ± 19.14
**Weight (kg)**		73.47 ± 56.91	81.88 ± 51.00	67.92 ± 54.48
**Body Mass Index (kg/m^2)**		25.31 ± 18.00	25.83 ± 14.76	24.96 ± 19.83
**Waist Circumference (cm)**		88.46 ± 47.79	91.94 + 43.14	86.16 ± 49.50
**Hip Circumference (cm)**		102.66 ± 33.18	102.89 ± 26.10	102.51 ± 37.17
**Waist-to-hip ratio**		0.857 ± 0.240	0.890 ± 0.234	0.835 ± 0.222

C. Demographic and anthropometric characteristics for NHANES (National Health and Nutrition Examination Survey) Dataset (limited to ages between 18 and 60). Data are X ± 3SD.

	**All**	**Men**	**Women**
**Total Number**	3197	1509	1688
**Height (cm)**	167.29 ± 30.09	174.54 ± 22.93	160.80 ± 21.07
**Weight (kg)**	83.65 ± 70.89	89.80 ± 67.43	78.15 ± 69.84
**Body Mass Index (kg/m2)**	29.78 ± 22.88	29.37 ± 19.83	30.14 ± 25.26
**Waist Circumference (cm)**	98.92 ± 54.16	100.72 ± 51.44	97.32 ± 56.05
**Hip Circumference (cm)**	107.01 ± 45.01	104.71 ± 37.40	109.06 ± 50.08
**Waist-to-hip ratio**	0.921 ± 0.245	0.957 ± 0.229	0.889 ± 0.216

10. Qualitative Comparisons Between Predicted and Ground Truth Meshes

Supplementary Figure 5. Qualitative comparisons for men. Qualitative comparisons of the SMPL mesh predicted by MeasureNet and state-of-the-art approaches for 3D human shape and pose estimation in men[1-3]. Images correspond to results presented in Table 3. The ground truth (GT) mesh is shown in the left.

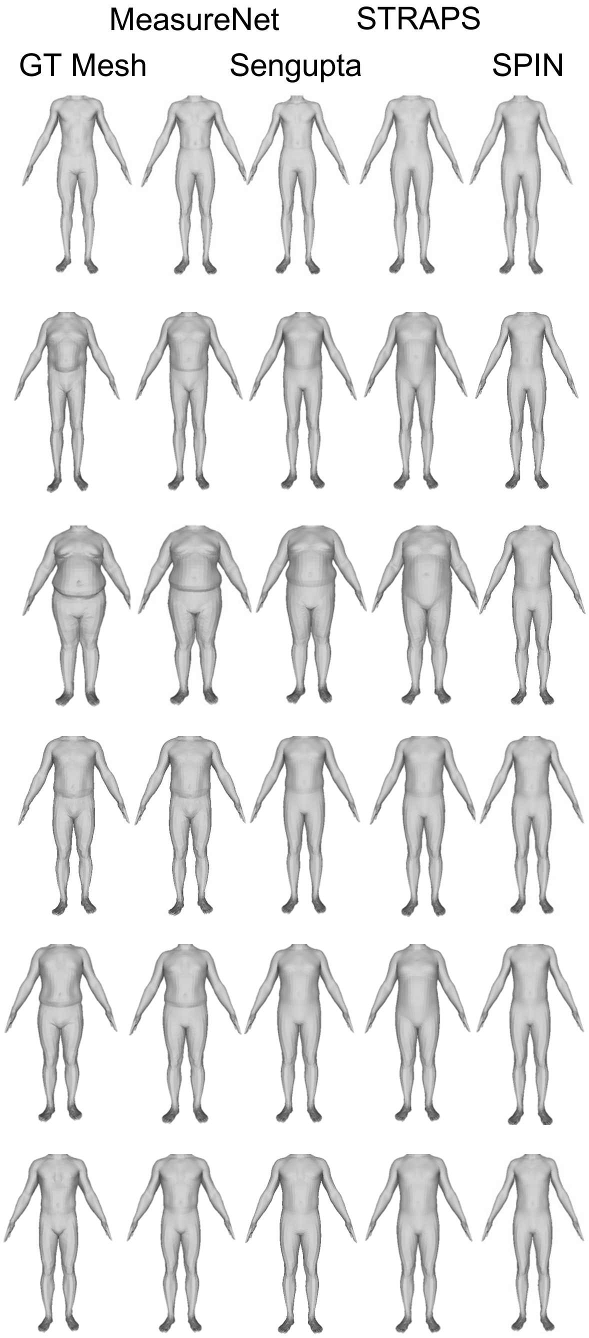

Supplementary Figure 6. Qualitative comparisons for women. Qualitative comparisons of the SMPL mesh predicted by MeasureNet and state-of-the-art approaches for 3D human shape and pose estimation in women[1-3]. Images correspond to results presented in Table 3. The ground truth (GT) mesh is shown in the left.

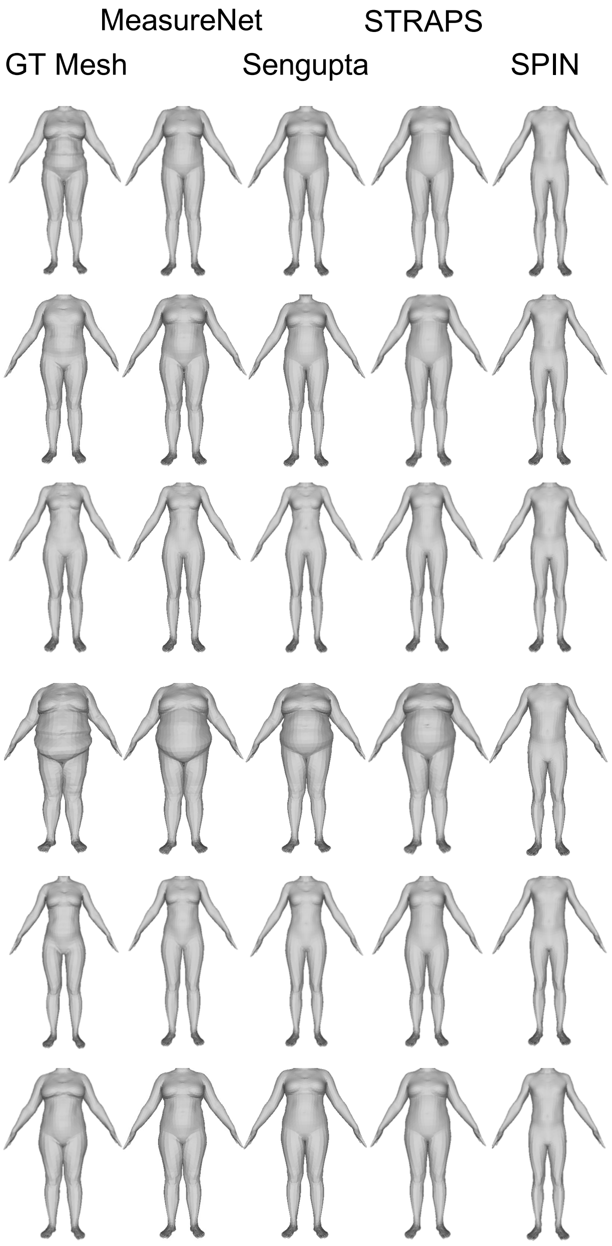

11. Noise Distributions

Supplementary Figure 7. Noise distributions in self-measurements. The differences between two measurements ($meas_1$ and $meas_2$) taken consecutively by the same user using a tape-measure are shown in red using histograms. We also fit a Gaussian curve (shown in black) on the resulting histogram to estimate the standard deviation of the noise. WHR is the waist to hip circumference ratio.

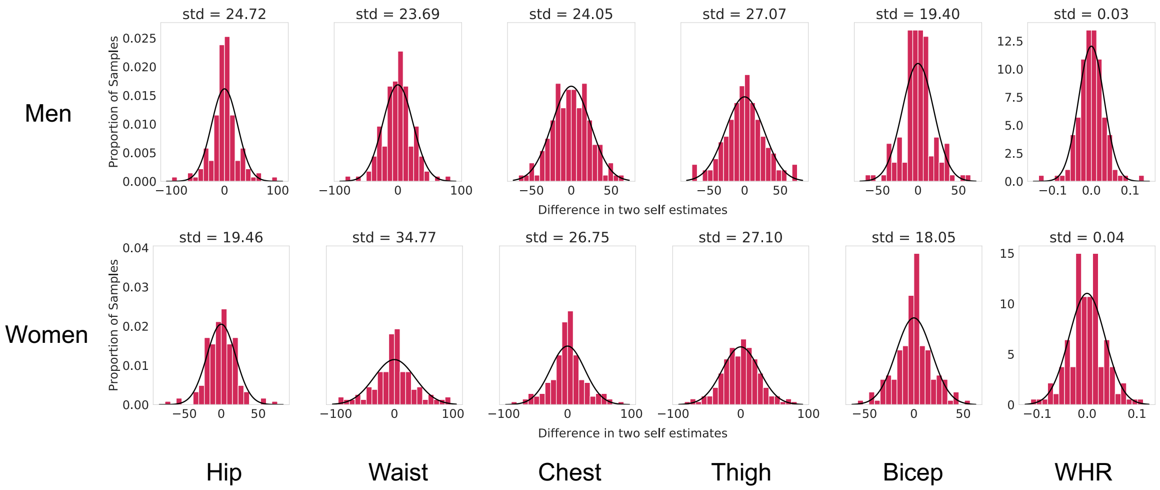

Supplementary Figure 8. Noise distributions in tape measurements taken by a trained staff member. The differences between two measurements ($meas_1$ and $meas_2$) taken consecutively by the same staff member using a tape-measure are shown in red using histograms. We also fit a Gaussian curve (shown in black) on the resulting histogram to estimate the standard deviation of the noise. WHR is the waist to hip circumference ratio.

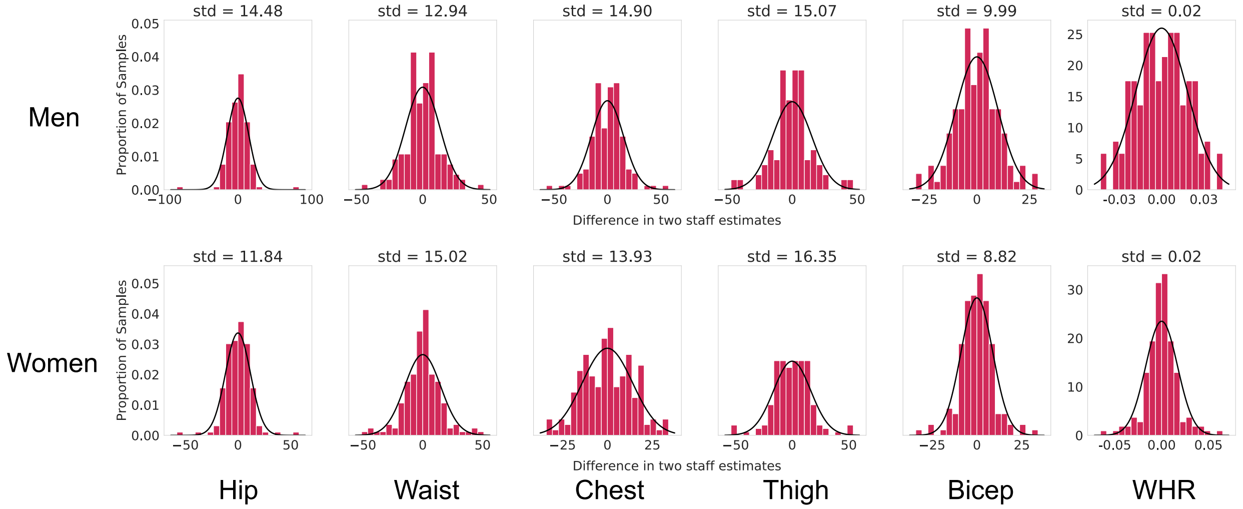

Supplementary Figure 9. Noise distributions in MeasureNet predictions. The differences between two predictions ($meas_1$ and $meas_2$) given the images taken consecutively by the same user are shown in red using histograms. We also fit a Gaussian curve (shown in black) on the resulting histogram to estimate the standard deviation of the noise. WHR is the waist to hip circumference ratio.

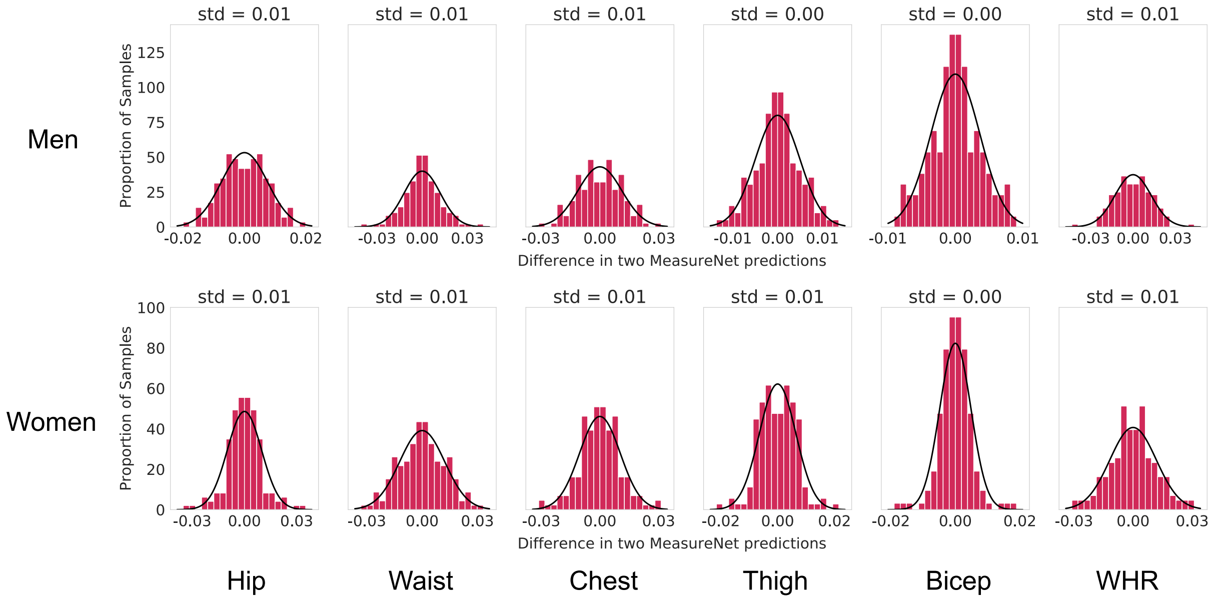

Supplementary References

1. Kolotouros N, Pavlakos G, Black MJ, Daniilidis K. Learning to reconstruct 3d human pose and shape via model-fitting in the loop. In Proceedings of the IEEE International Conference on Computer Vision, 2019.
2. Sengupta A, Budvytis I, Cipolla R. Hierarchical Kinematic Probability Distributions for 3D Human Shape and Pose Estimation from Images in the Wild. In International Conference on Computer Vision, October 2021.
3. Sengupta A, Budvytis I, Cipolla R. Synthetic training for accurate 3d human pose and shape estimation in the wild. In British Machine Vision Conference (BMVC), September 2020.

4. Kendall A, Gal Y, Cipolla R. Multi-task learning using uncertainty to weigh losses for scene geometry and semantics. *Proceedings of the 2018 IEEE Conference on Computer Vision and Pattern Recognition (CVPR)*, 7482-7491 (2018).
